# Comparison of the Efficacy of a Diabetes Education Programme for Type 1 Diabetes (PRIMAS) in a Randomised Controlled Trial Setting and the Effectiveness in a Routine Care Setting: Results of a Comparative Effectiveness Study

**DOI:** 10.1371/journal.pone.0147581

**Published:** 2016-01-22

**Authors:** Dominic Ehrmann, Nikola Bergis-Jurgan, Thomas Haak, Bernhard Kulzer, Norbert Hermanns

**Affiliations:** 1 Research Institute of the Diabetes Academy Mergentheim (FIDAM), Johann Hammer-Str. 24, 97980, Bad Mergentheim, Germany; 2 Diabetes Centre Mergentheim, Diabetes Clinic, Theodor-Klotzbuecher-Str. 12, 97980, Bad Mergentheim, Germany; 3 Otto-Friedrich-University of Bamberg, Department of Clinical Psychology and Psychotherapy, Bamberg, Germany; Weill Cornell Medical College Qatar, QATAR

## Abstract

**Background:**

The effectiveness of an intervention in clinical practice is often reduced compared to the efficacy demonstrated in a randomised controlled trial (RCT). In this comparative effectiveness study, the RCT-proven efficacy of a diabetes education programme for type 1 diabetic patients (PRIMAS) was compared to the effectiveness observed in an implementation trial (IT) under routine care conditions.

**Methods:**

75 patients with type 1 diabetes received PRIMAS through an RCT, whereas 179 patients were observed in an implementation trial. Baseline characteristics and treatment outcomes at the 6-month follow-up (improvement of HbA1c, hypoglycaemia problems, and diabetes-related distress) were compared.

**Results:**

At baseline, the type 1 diabetic patients in the RCT had a significant longer diabetes duration (18.7±12.3 vs. 13.8±12.7 yrs., p = .005), lower self-efficacy scores (21.9±4.7 vs. 23.7±6.1, p = .02) and a greater number of diabetes complications (0.8±1.3 vs. 0.4±0.9, p = .02). After 6 months, PRIMAS achieved comparable effects under RCT and implementation trial conditions, as demonstrated by improvement in HbA1c (-0.36%±1.1 vs. -0.37±1.2; Δ -0.01, 95% CI -0.33 to 0.31) and hypoglycaemia unawareness (-0.5±1.4 vs. -0.3±1.4; Δ 0.18, 95% CI -0.21 to 0.57). The likelihood of clinical improvement did not depend on the trial setting (RCT vs. IT: OR 1.18, 95% CI 0.60 to 2.33). The participants with worse glycaemic control (OR 1.40, 95% CI 1.02 to 1.92), hypoglycaemia problems (OR 2.13, 95% CI 1.53 to 2.97) or elevated diabetes distress (OR 1.40, 95% CI 1.03 to 1.89) had a better chance of clinical improvement.

**Conclusions:**

The effectiveness of PRIMAS under routine care conditions was comparable to the efficacy demonstrated in the RCT. Clinical improvement was independent of the setting in which PRIMAS was evaluated. The PRIMAS education programme for type 1 diabetes can be delivered under conditions of routine care without a loss of effectiveness, compared to its original evaluation in an RCT.

## Introduction

Structured diabetes education has been an integral part of the treatment of type 1 diabetes for decades [1–6]. The objectives of structured diabetes education are multifaceted as they range from improving glycaemic control and problems with hypoglycaemia to reducing diabetes-related emotional distress [7]. In summary, diabetes education aims to empower people with type 1 diabetes to manage their diabetes treatment (especially intensive insulin therapy) and the emotional challenges associated with their chronic disease by themselves [8]. Considering these different objectives, diabetes education and treatment programmes are complex interventional measures.

In addition to the transfer of knowledge and skills, the enhancement of diabetes self-management is an important component of modern diabetes education programmes. Therefore, diabetes education programmes should include various tools or components that enable active self-management, such as discussion of individual attitudes and barriers and how to cope with perceived problems of living with diabetes as well as practicing skills to deal with the challenges of diabetes in daily life.

Adding to the complexity, the outcomes of structured diabetes education for type 1 diabetes not only depend on education itself but also on several circumstantial factors. These include the diabetes regimen, the knowledge and skills of the health care professionals who deliver the diabetes education, and the motivation of the patients to participate in the diabetes education and to implement new skills into their daily routine [9].

Therefore, structured diabetes education and treatment programmes not only are considered to be complex interventions but are also complex to evaluate as their efficacy relies on a number of different, partially fixed circumstantial factors. The multiple components of diabetes education cannot be tested separately. In addition, other design requirements that relate to the evaluation of pharmacological interventions, such as double-blind delivery of the intervention, are not possible in the evaluation of diabetes education.

For these reasons, the U.K. Medical Research Council [10–12] established an algorithm for the development and evaluation of complex interventions. This algorithm was applied to the development and evaluation of diabetes education programmes by Mühlhauser & Berger [13]. The algorithm describes how complex intervention should be developed; theoretical considerations should be considered first, and then a modelling phase, feasibility studies, and a randomised controlled trial (RCT) should follow to demonstrate the overall efficacy of the complex intervention. In addition to the primary test of the efficacy of structured diabetes education programmes through an RCT, evaluation of the implementation of new diabetes education programmes in clinical care settings should be a key feature of the evaluation of such complex interventions.

According to many health care research trials, there is a gap between the efficacy in RCTs and the effectiveness in clinical practice [14–16]. Therefore, implementation trials (ITs) conducted as part of comparative effectiveness research [17] that compare the efficacy of diabetes education conducted under more strict conditions of an RCT with the effectiveness of education conducted under less strict conditions of routine care are necessary. Possible differences between the results obtained using an RCT and IT could be due to differences in sample composition related to the stricter inclusion and exclusion criteria of RCTs. Additionally, the implementation of a structured intervention in routine care is frequently associated with reduced standardisation of the intervention due to differences in the clinical settings in which the complex intervention is used.

We recently developed a treatment and education programme for people with type 1 diabetes (PRIMAS) that aimed to improve glycaemic control by enhancing self-management. PRIMAS was evaluated in an RCT, and its efficacy was demonstrated [18].

Following the suggestions of Mühlhauser & Berger [13], an IT was conducted to evaluate the effectiveness of PRIMAS under routine care conditions.

The aim of this comparative effectiveness study was to compare the baseline characteristics and treatment outcomes of PRIMAS participants when the programme was conducted under the conditions of an RCT or in a routine care setting in an IT. Possible differences between the RCT and IT refer to the selection of participants, the medical problems that indicate a participation in a structured diabetes education programme, and treatment outcomes.

## Materials and Methods

### Development of the PRIMAS intervention

PRIMAS, a new structured diabetes education and treatment programme for type 1 diabetes, was developed according to the criteria established by the U.K. Medical Research Council. During the theoretical phase, the shift from more knowledge-driven educational concepts to more self-management-oriented educational concepts greatly influenced the conceptualisation of PRIMAS. Furthermore, PRIMAS also reflects the change from educational concepts aimed at optimal compliance with treatment recommendations to a concept that is focused on empowering people to actively participate in treatment decisions. New technologies, such as advances in continuous glucose monitoring as well as new insulin and CSII devices, were also an important aspect in the conceptualisation of the PRIMAS programme.

During the modelling phase, formative evaluation techniques were applied to introduce and discuss the new educational material with diabetologists and diabetes educators. Pilot testing of the new educational material was carried out in certain diabetologist practices to improve and refine the concepts and material.

During the evaluation phase, an RCT was conducted to investigate the impact of PRIMAS on glycaemic control and a variety of other outcome variables compared to that of an established programme for type 1 diabetes. The results of the RCT were published in 2013 [18].

### Subjects

The eligibility criteria for the RCT and IT are reported in Table 1. In the RCT, the recruitment of patients was based on the inclusion criteria, whereas in the IT sample, recruitment was based on clinical indication, as defined by the treating physician. These indications typically include a lack of previous diabetes education, considerable passage of time since the last education, suboptimal control (elevated HbA1C levels or hypoglycaemia problems) despite optimised treatment, or psychosocial problems that impair coping with diabetes. The IT clearly had less inclusion criteria than the RCT. With the exception of a duration of type 1 diabetes of more than 1 month, there were no specific inclusion criteria for the IT. Specifically, there were no age limits or eligibility criteria based on glycaemic control or BMI in the IT. Patients in both trials had to possess the language skills to follow diabetes education and the need for diabetes education according to the assessment of the treating physician.

**Table 1:** Inclusion criteria for the RCT and IT.

RCT	IT
type 1 diabetes	type 1 diabetes
age ≥18 and ≤75 years	-
diabetes duration >1 month	diabetes duration >1 month
BMI >20 and <40 kg/m^2^	-
HbA1c ≥7.0% and ≤13.0%	-

Exclusion criteria for both the RCT and IT included current psychological or psychiatric disorder (under treatment), dementia or severe cognitive impairment, severe somatic disease (that would prevent regular participation in the education course), and pregnancy.

### Trial settings

There were 2 measurement points in the RCT and IT. The first measurement took place at baseline prior to the start of the education course—in case of the RCT the measurement took place before randomisation. The second measurement was conducted 6 months after the end of the education course. Both measurements were conducted at the site of each patient’s diabetes care practice. The timing of the follow-up assessment was in line with current routine care for people with type 1 diabetes; routine care guidelines recommend visits every 3 months for HbA1c measurement. In the IT setting, patients were asked to complete questionnaires at their second routine care visit after participation in the PRIMAS course. Furthermore, a blood sample was drawn for the measurement of HbA1c in a central laboratory (the same laboratory as that used in the RCT). Hence, patients of the IT did not receive care that differed from the usual care of people with type 1 diabetes. In the RCT, specialised diabetes care practices at the secondary care level (n = 23), including diabetologists and certified diabetes educators, were in charge of the conduction of PRIMAS. The duration of the PRIMAS course (12 lessons) in the RCT was predefined, with 2 lessons each week. In the IT, primary (n = 21) and secondary care level (n = 21) practices conducted PRIMAS. No predefined course duration was given in the IT. In addition, a high level of standardisation and fidelity was established in the RCT, such as monitoring the conduction of PRIMAS as well as the conduction of the study. Practices in the IT did not receive any special training in conducting PRIMAS.

The RCT began in September 2010 (first patient in) and ended in January 2012 (last patient out). The IT began in September 2012 (first patient in) and ended in November 2013 (last patient out).

### Outcome measures

The reduction of HbA1c within 6 months of the termination of the intervention was the primary outcome of the RCT [18]. For the present analysis, the HbA1c reduction achieved in the RCT was compared to the reduction achieved in the IT. In both trials, HbA1c was measured in the same central laboratory using the HPLC method (normal range: 4.3% to 6.1%; 23.5 to 43.2 mmol/mol).

Other outcome measures were assessed as follows:

Diabetes-related distress was assessed using the German version of the Diabetes Distress Scale (DDS). The DDS is a well validated and widely applied 17-item self-report scale that evaluates the current level of diabetes-related emotional distress for both type 1 and type 2 diabetes [19].

Empowerment was measured using the German version of the Diabetes Empowerment Scale, which was developed by Anderson et al. [20].

A German version of the General Self-efficacy Scale [21] was used to assess self-efficacy.

Hypoglycaemia awareness was assessed using the German version [22] of the Hypoglycaemia Awareness Scale developed by Clarke et al. [23]. This scale ranges from 0 (maximum hypoglycaemia awareness) to 7 (minimum hypoglycaemia awareness), with a score of 4 or higher suggesting reduced hypoglycaemia awareness.

In addition to defining singular primary and secondary outcomes, we defined a combined outcome. For this combined outcome, we used baseline data and defined possible clinical problems. Three problems were defined: (I) Suboptimal glycaemic control was defined by a HbA1c > 7.5% [24]; (II) Hypoglycaemia problems were defined as having experienced severe hypoglycaemia during the past 12 months (third party assistance needed) or a score of 4 or higher on the Hypoglycaemia Awareness Scale (indicating hypoglycaemia unawareness); and (III) Psychosocial problems due to living with diabetes were defined by a score on the DDS of 3 or higher [25]. In summary, participants could be classified with up to three clinical problems that indicate a need for structured diabetes education (suboptimal glycaemic control, and/or hypoglycaemia problems, and/or psychosocial problems).

Demographic and other medical data were retrieved from medical records via case-report-forms.

### Clinical ethics

Both trials were approved by the ethics committee of the German Psychological Association (approval number: RCT: NH 062010, IT: NH 072012). All patients included in the trials provided written informed consent.

### Statistical analysis

In this comparative effectiveness study, the baseline characteristics of the participants in the IT and RCT trials were compared. The outcomes of PRIMAS under routine care conditions and the conditions of the RCT were also compared.

The difference between baseline and 6-month follow-up for HbA1c and the other secondary outcome parameters were the dependent variables. Condition (delivery in the RCT or IT) was the independent variable. Student t-tests were used for parametric data, and Chi-Square tests were used for categorical data.

A multivariate logistic regression analysis was performed with clinical improvement as the dependent variable. Clinical improvement was defined as improvement in glycaemic control, and/or hypoglycaemia awareness, and/or psychosocial problems. The independent variable of interest was trial setting (RCT vs. IT). Additional baseline characteristics were also included, such as gender, insulin regimen (insulin pump [CSII] therapy vs. multiple daily insulin injections [MDI]) and z-scores for age, diabetes duration, BMI, HbA1c, hypoglycaemia awareness, and diabetes-related distress.

No substitutions of missing data were made. The statistical software programme SYSTAT 12.0 (Systat Software, Inc.; Chicago, IL) was used for the statistical analysis.

## Results

In the IT, 179 people with type 1 diabetes from 42 practices were monitored for 6 months. The data of these participants were compared with those of the 75 participants from the 23 practices of the RCT who received PRIMAS [18]. The baseline characteristics of the sample that received PRIMAS in the RCT and IT are described in Table 2.

**Table 2:** Baseline characteristics of the patients in the RCT and IT.

Characteristic	RCT (n = 75)	IT (n = 179)	p
Age—years (±SD)	45.1 (±13.5)	43.6 (±13.6)	.412
% female gender—n (%)	38.7%	46.9%	.228
Years of education—mean (±SD)	11.2 (±3.1)	11.1 (±3.0)	.916
% with migration background	2.7%	5.6%	.319
Mean diabetes duration—years (±SD)	18.8 (±12.3)	13.8 (±12.7)	.**005**
BMI—kg/m^2^ (±SD)	26.5 (±4.6)	26.0 (±4.7)	.435
HbA1c—% (±SD)—*mmol/mol (±SD)*	8.3 (±1.1) *67 (±12*.*0)*	7.9 (±1.4) *63 (±15*.*3)*	.056
# insulin injections—n (±SD)	5.2 (±1.4)	5.3 (± 1.5)	.754
IU/KG—mean (±SD)	0.66 (±0.34)	0.60 (±0.29)	.162
% with CSII therapy	25.3%	14.7%	.**044**
# of blood glucose self-tests per day (±SD)	4.7 (±1.6)	5.1 (±1.7)	.096
Late complications—n (±SD)	0.8 (±1.3)	0.5 (±0.9)	.**021**
Unawareness score (Range 0–7)	1.8 (±1.7)	1.6 (±1.5)	.409
% with severe hypoglycaemic episodes in the 12 months	14.9%	21.3%	.245
% with previous structured diabetes education	85.1%	74.3%	.061
Diabetes Distress Scale (Range 0–5)	1.3 (±1.0)	1.1 (±0.8)	.054
Empowerment (Range 0–33)	24.7 (±6.0)	24.5 (±5.7)	.836
Self-efficacy (Range 0–30)	21.9 (±4.7)	23.7 (±6.1)	.**024**

### Baseline characteristics

Participants of the RCT and IT had similar educational statuses and migration backgrounds. The proportion of CSII-patients and the number of daily insulin injections in the patients with MDI treatment indicate that the participants were already performing intensive insulin therapy. The RCT included significantly more patients on CSII therapy. Daily blood glucose measurements and insulin demand in the RCT and IT were highly comparable. Furthermore, hypoglycaemia unawareness and the proportion of people with severe hypoglycaemia (third party assistance needed for recovery) did not differ between the RCT and IT.

The patients participating in the RCT had a significantly longer diabetes duration than the participants in the IT. The participants of the RCT reported lower self-efficacy with respect to the management of diabetes. The baseline results indicate that the participants in the RCT had more long-term complications than the participants in the IT. Furthermore, the participants in the RCT were more distressed and had higher HbA1c levels than the participants in the IT, but these differences were only marginally significant (p < .10).

In a further analysis, we investigated the possible problems that indicate a need toparticipate in a diabetes education course, distinguishing between people with suboptimal glycaemic control (HBA1c >7.5%), hypoglycaemia awareness issues (experience of a severe hypoglycaemic episode during the past 12 months or a hypoglycaemia unawareness score ≥ 4) or psychosocial problems due to living with diabetes (diabetes distress scale score ≥ 3).

**Fig 1:**
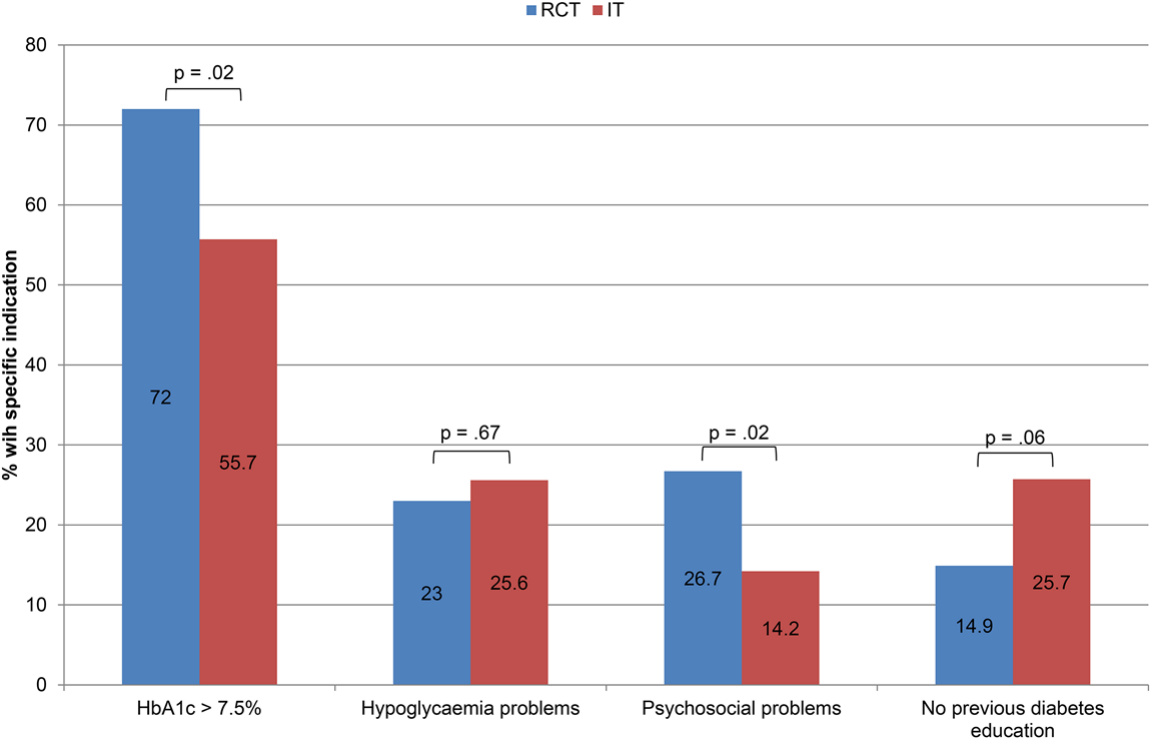
Prevalence of clinical problems (suboptimal glycaemic control, hypoglycaemia problems, and psychosocial problems) at baseline in the RCT and IT participants.

Fig 1 provides an overview of the clinical problems of participants of diabetes education in the RCT and IT setting. A greater number of the participants in the RCT had HbA1c levels above 7.5% and reported psychosocial problems.

### Outcomes of diabetes education

First, the overall effect of PRIMAS was tested by comparing baseline and follow-up measurements for both trials combined (RCT and IT sample). Six months after participating in PRIMAS, significant improvement in glycaemic control (HbA1c: -0.36, 95% CI -0.22 to -0.51, p < .01), hypoglycaemia awareness (unawareness score: -0.36, 95% CI -0.20-to -0.58, p < .01), diabetes-related distress (DDS score: -0.18, 95% CI -0.09 to -0.28, p < .01), empowerment (empowerment score: +2.7, 95% CI 1.7 to 3.7, p < .01) and self-efficacy (self-efficacy score: +1.1, 95% CI 0.3 to 1.9, p = .01) was observed.

### Differences in treatment outcomes: RCT vs. IT

The specific outcomes of participants in the RCT and IT are reported in Table 3. After 6 months, the outcomes of the RCT were highly comparable to those of the IT. The difference in the reduction of HbA1c was minimal and far from being significant. Differences in the improvement of hypoglycaemia unawareness, empowerment, and self-efficacy between the RCT and IT were also not statistically significant. Therefore, the delivery of PRIMAS in a routine care setting was not substantially less efficacious than its delivery during the RCT. The only exception was a significantly greater reduction of diabetes-related distress in the RCT. However, since significant differences in diabetes-related distress were observed at baseline, the analysis was adjusted for baseline differences of the DDS scores. The baseline-adjusted between-group difference did not significantly differ between the RCT and IT (Δ -0.03, 95% CI -0.19 to 0.12, p = .68).

**Table 3:** Differences between the outcome effects (baseline– 6-month follow-up) for the RCT and IT.

Outcome	RCT (n = 75)	IT (n = 179)	Between-group difference (95% CI)	p*	Effect size of difference (95% CI)
HbA1c—% (±SD)—*mmol/mol (±SD) (n missing = 19)*	-0.36 (±1.05) *- 4*.*0 (±11*.*5)*	-0.37 (±1.19) *- 4*.*0 (±13*.*0)*	-0.01 (-0.33–0.31)	.959	0.01 (-0.29–0.31)
Unawareness score *(n missing = 38)*	-0.51 (±1.42)	-0.33 (±1.38)	0.18 (-0.22–0.57)	.373	0.13 (-0.16–0.42)
Diabetes Distress Scale *(n missing = 34)*	-0.34 (±0.75)	-0.11 (±0.67)	0.23 (0.04–0.43)	.**021**	0.36 (0.06–0.67)
Empowerment *(n missing = 35)*	+2.61 (±5.94)	+2.84 (±8.26)	-0.23 (-2.36–1.90)	.831	-0.03 (-0.34–0.27)
Self-efficacy *(n missing = 35)*	+1.40 (±3.56)	+0.93 (±7.34)	0.46 (-1.32–2.24)	.609	0.08 (-0.23–0.39)

* Between group differences

Table 3 also depicts the effect sizes of the differences in the outcomes achieved in the RCT compared to the IT. Effect sizes ranging from -0.03 to 0.36 standard deviations are indicative of rather small to moderate effect sizes.

### Impact of trial setting on clinical improvement

**Fig 2:**
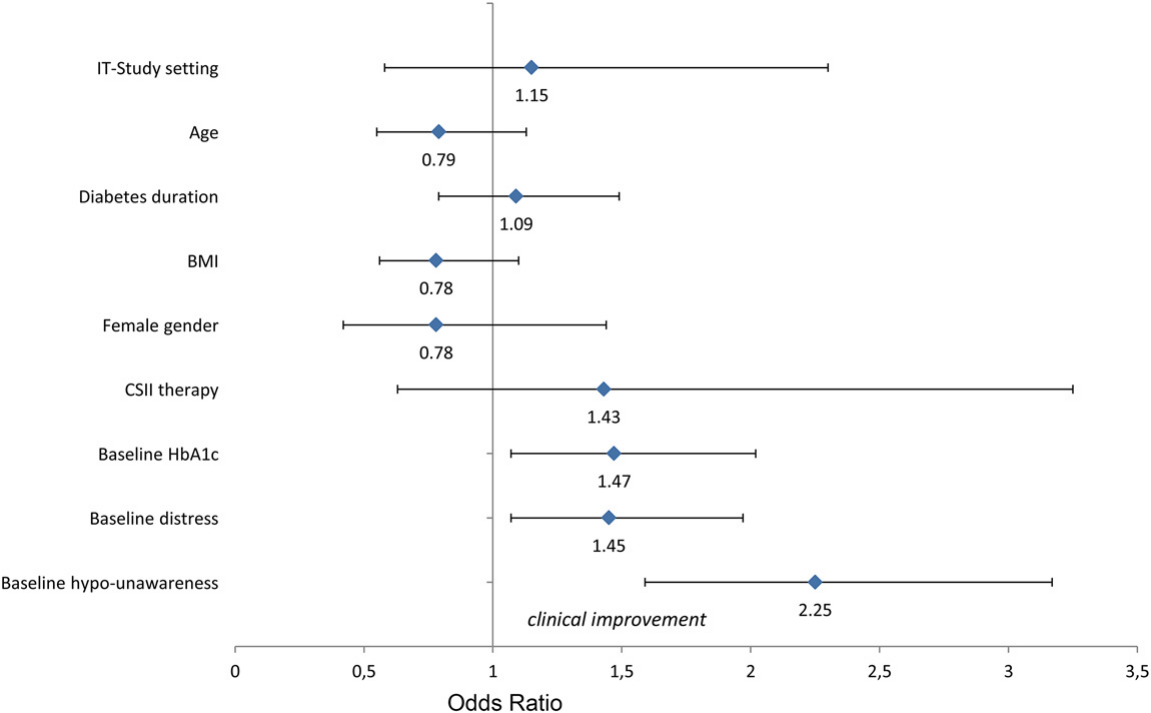
Odds ratios for clinical improvement in glycaemic control, hypoglycaemia awareness and/or diabetes-related distress (continuous variables: odds ratio per 1 standard deviation increase). * p < .05.

In an additional analysis, the impact of the trial setting (RCT vs. IT) on clinical improvement at the 6-month follow-up was analysed. Clinical improvement was defined as an improvement in at least one of the clinical outcomes, glycaemic control, hypoglycaemia problems or elevated diabetes-related distress. A multivariate logistic regression analysis with trial setting (RCT vs. IT) as the independent variable was performed, and baseline sample characteristics were controlled for (see Fig 2). The multivariate regression model did not show a significant effect of trial setting (RCT vs. IT) on the odds ratio to achieve clinical improvement in any of the clinical outcomes. Furthermore, neither the baseline demographic variables, such as age or gender, nor the medical variables, such as BMI or treatment (CSII vs. MDI), had a significant effect. Interestingly, higher HbA1c values, higher hypoglycaemia unawareness scores and higher diabetes distress scores increased the likelihood of clinical improvement 6 months after diabetes education, regardless of trial setting (RCT vs. IT).

## Discussion

This comparative effectiveness analysis of the PRIMAS education programme for type 1 diabetes under RCT and routine care conditions revealed different sample composition between the RCT and IT trials but comparable clinical outcome effects. The setting of the delivery of PRIMAS (RCT vs. IT) did not have a significant impact on clinical improvements when baseline demographic and medical variables were controlled for.

### Baseline characteristics

The participants in the RCT setting had a significantly longer diabetes duration, more complications, lower self-efficacy, marginally more diabetes-related distress, and higher HbA1c levels than the participants in the IT setting. A selection bias may have influenced the sample compositions. Patients with a more complicated course of their diabetes seem to be easier to motivate to participate in an RCT, whereas patients with a less complicated course of their diabetes may rather volunteer for an observational trial under routine care conditions [24].

The medical problems used as indicators for participation in diabetes education, such as suboptimal glycaemic control or elevated diabetes-related distress, were significantly more frequent in the RCT sample than in the IT sample. The finding regarding glycaemic control may have been due to the inclusion criterion of HbA1c > 7.5% [18]. The higher proportion of participants with elevated diabetes-related distress observed in the RCT could be attributable to the more complicated course of their diabetes compared to that of the IT participants (i.e., more late complications).

The observed baseline differences are in line with observations made in other comparative effectiveness studies, which showed that people who seek treatment in observational trials are younger, have more access to health care and have a better prognosis than people who are not seeking treatment [25].

### Outcomes of diabetes education

Diabetes education with PRIMAS led to a 0.36-percentage-point reduction in HbA1c when both trials were combined. The overall extent of improvement may be less than in previous type 1 diabetes education trials [2–5]; however, it has to be taken into account that in early type 1 diabetes education trials, the participants were switched from a conventional insulin regimen to an intensive insulin treatment, making it difficult to separate the effect of diabetes education from the impact of the insulin regimen change. In the present analysis, the baseline characteristics suggest that the vast majority of the participants were already on an intensive insulin therapy regimen and had remarkably better glycaemic control at baseline than the participants in the diabetes education trials cited above. This may have limited the potential for improvement in glycaemic control in both PRIMAS trials. However, a similar effect regarding HbA1c-reduction was reported in the implementation trial of the DAFNE programme [26]. After 6 months, the authors found a reduction in HbA1c of 0.3%. Interestingly, baseline HbA1c for routine care was 8.5% compared to the 9.4% found in the initial DAFNE RCT [2–5]. Regarding baseline HbA1c and achieved reduction in HbA1c, the results from DAFNE in a routine care setting are highly comparable to the results obtained in both PRIMAS trials.

Remarkably, although PRIMAS was able to improve glycaemic control, there was also a significant improvement in hypoglycaemia awareness in both trials. This is in line with previous findings that improvements in glycaemic control within education trials do not necessarily increase the risk for hypoglycaemia [27]. In addition to metabolic improvements, the participants of the RCT and IT also reported lower diabetes-related distress and higher empowerment and self-efficacy. This can be interpreted as an indication that diabetes self-management in people with type 1 diabetes [8] was positively affected by PRIMAS; a similar effect has been shown for type 2 diabetes in meta-analyses and reviews [28–30].

### Differences in treatment outcomes: RCT vs. IT

Comparing the HbA1c reduction for the RCT and IT revealed no substantial differences between the settings. The mean difference was only 0.01 percentage points. The 95% confidence interval of this difference did not exceed the threshold of 0.4 percentage points, which is a commonly used threshold to determine clinical non-inferiority with regard to improvements in glycaemic control [31]. Thus, the results of the IT indicate that with respect to glycaemic control, PRIMAS was equally effective when delivered in a routine care setting as when it was delivered in the RCT [18].

In addition, the comparisons of the other outcomes also indicate that the effects of PRIMAS at the 6-month follow-up that were achieved in the IT were highly comparable to those achieved in the RCT. Only the reduction of diabetes-related distress was significantly greater in the RCT than in the IT. At baseline, however, the participants of the RCT already exhibited significantly higher distress scores than the participants of the IT. With the exception of the reduction in diabetes-related distress, the effect sizes of the differences between the RCT and IT were rather small [32], which corroborates the notion of equal effectiveness. While the baseline difference may account for the greater reduction of distress in the RCT, a likely explanation may also be that the elements of PRIMAS that triggered the improvement in distress were less effective in the IT. This may be attributable to the better training of RCT practices prior to study start. The better training was due to stricter study SOPs for the delivery of the intervention compared to routine care.

### Impact of trial setting on clinical improvement

The results of the multivariate logistic regression analysis showed that clinical improvement in at least one of the three clinical problems (suboptimal glycaemic control, hypoglycaemia problems, or elevated distress) was independent of the setting in which PRIMAS was conducted (RCT vs. IT). Thus, important clinical problems can be addressed and significantly improved by the PRIMAS programme. Improvement of those problems could be achieved not only in a controlled RCT environment but also in a routine care setting. Furthermore, the effects of PRIMAS were not limited to a specific patient group, as demographic variables were not significant in the logistic regression analysis.

Not only was there a difference in study setting, there was also a difference in the degree of specialization of the practices that participated in the RCT and IT. We therefore analysed whether care level had an impact on the clinical improvement achieved through PRIMAS. Care level was included in the logistic regression analysis, but it was not a significant predictor of clinical improvement and did not substantially alter the odds ratios or the significance of the remaining variables (data not shown). Hence, this result indicates that PRIMAS is equally effective at the primary and secondary care level.

### Limitations and strengths

When interpreting the results of the study, the following limitation should be considered. This study was a post hoc analysis of results from an RCT and an IT. Although the results suggest equal effects on key outcomes except diabetes-related distress, the design was not a formal non-inferiority study [33].

However, comparison of efficacy in an RCT and effectiveness in an IT can provide valuable insight into the transfer of a diabetes education programme from an artificially controlled research setting into clinical practice. The IT closely mirrors the clinical practice of diabetes education with PRIMAS, as it did not include strict eligibility criteria or predefine how the education course should be conducted. In addition, not only specialised diabetologists took part in the IT but also less-specialised practices at the primary care level. All in all, the generalisability of this comparative effectiveness study can be considered high because not only did the IT include a wider range of patients but a more representative range of practices was also used to conduct PRIMAS.

### Conclusion

In summary, this comparative effectiveness analysis showed that the PRIMAS education programme for type 1 diabetes can be delivered under conditions of routine care without a loss of effectiveness compared to that found during its original evaluation in an RCT.

## Supporting Information

S1 Dataset Comparison of PRIMAS effectiveness. (XLSX)
